# Book Reviews

**Published:** 1890-08

**Authors:** 


A NEW MEDICAL DICTIONARY—Includes all the Words and Phrases used in Medicine, with their Proper Pronunciation and Definitions, Based on Recent Medical Literature. By George M. Gould, B. A., M. D., Ophthalmic Surgeon to the Philadelphia Hospital, and Clinical Chief Opthalmological Department German Hospital, Philadelphia. Published by P. Blakiston, Son & Co., Philadelphia, Pa.

This is a very valuable work to the busy practitioner, as well as the student. Dunglison's may well be superseded by this, since the new words which have become technical may have to be found in their full orthography and orthoepy.

Being handsomely and strongly bound, it will stand the usage that such a work has to undergo. The attention devoted to bacteriology, anatomy and other important details is alone sufficient to recommend the book to the profession.

The artistic manner in which the volume is gotten up reflects much credit upon the publishers, and nothing seems to be wanting in this respect but a little closer attention given to proof-reading, as the mistakes seem to be more of a typographical than a literary character.

---

THE HOMILETIC REVIEW for August gives no evidence that extreme heat is the order of the day. Its pages are as breezy and tonic as ever. Prof. Knox, of the German Theological Seminary, opens with an admirable and timely paper on Biblical Homiletics, which every preacher ought to read. Dr. Schodde follows with a valuable paper on Recent Researches in Bible Lands. Dr. A. T. Pierson discusses the Secrets of Pulpit Power. Dr. Sample, of New York, in a well considered and discriminating article, presents the subject of Responsibility for Belief. Of the sermons in the number it is sufficient to say they are by such able preachers as Dr. Thwing of Minneapolis, Whitley of Virginia, Dr. Withrow, of Chicago, Dixon of Baltimore, Dr. J. M. Ludlow, Dr. Wright of London, and Parker of China. The other departments, such as the Prayer-meeting Service, the Exegetical, the European by Dr. Stuckenberg, the English by Dr. Joseph Parker, the Miscellaneous and the Editorial, are each and all brim full of fresh and instructive thought on all the varied themes which specially interest our pastors and preachers.

Published by Funk & Wagnalls, 20 Astor Place, New York. $3.00 per year; single copies, 30 cents.

---

THE THERAPEUTIC APPLICATION OF PEROXIDE OF HYDROGEN.—Drevett Co., New York.

An interesting and scientific discussion on this important subject, and a book which will prove of value to every practitioner. The little book will be cheerfully furnished any physician on application.

---

THE DANGEROUS FRENCH STOVE.—The Paris Academy of Medicine has had its attention directed to the defective and dangerous form of portable stoves which are such a frequent source of carbonic oxide poisoning. M. Laucereaux and others considered the subject of so much importance to the public health as to require governmental supervision and control; others considered that it would only be necessary to inform the public of the dangers of this mode of heating. According to Laborde the presence of 1 part of carbonic oxide in 650 parts of air is injurious to life and may cause death. The advocates of supervision carried their point, and the Academy passed resolutions condemning the slow-draught or air-tight stove in nurseries, schools, colleges, and sleeping-rooms. It also called the attention of the authorities to the dangers of this mode of heating, and petitioned for remedial legislation.—Report of Dr. Prosper de Pietra Santa, in the *Satellite* for April, 1890.

